# Supplementary figures and images for: Evidence that human oral glucose detection involves a sweet taste pathway and a glucose transporter pathway
Source: PLoS One. 2021 Oct 6;16(10):e0256989. doi: 10.1371/journal.pone.0256989 (PMC8494309; doi:10.1371/journal.pone.0256989)

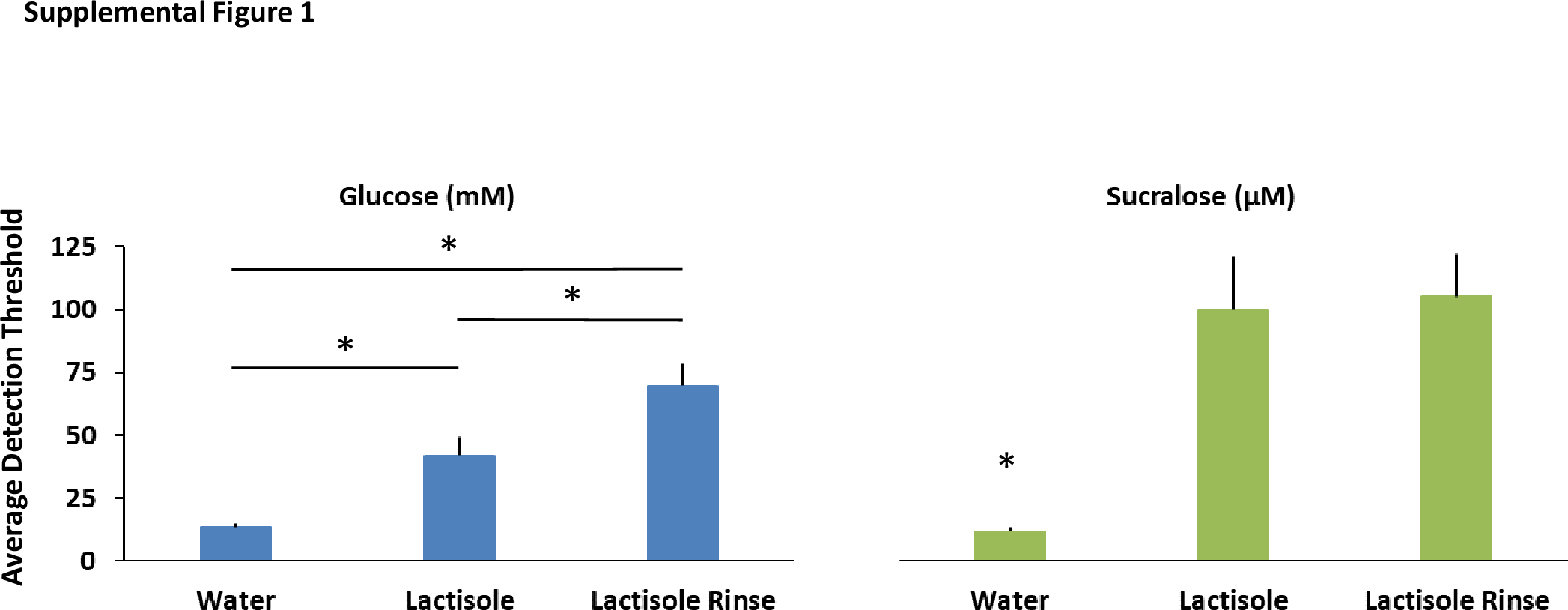

Supplement: S1 Fig — There are two charts. The chart on the left (blue bars) shows average detection thresholds for glucose (mM). The chart on the right (green bars) shows average detection thresholds for sucralose (μM). In each chart there are three bars. From left to right the first bar is the average detection threshold for the sweetener when presented against water. The second bar is the average detection threshold for the sweetener with the addition of 2mM lactisole. The third bar is the average detection threshold for the sweetener with 0.8mM lactisole rinses between samples. The lactisole rinses were to determine if the lactisole treatment gave rise to a “sweet water taste.” Twelve subjects were tested in all conditions in duplicate. The error bars are standard errors of the mean. * indicates a significant difference p < .001. (TIF) [file pone.0256989.s001.tif]
