# Supplementary material for: Evidence that human oral glucose detection involves a sweet taste pathway and a glucose transporter pathway
Source: PLoS One. 2021 Oct 6;16(10):e0256989. doi: 10.1371/journal.pone.0256989 (PMC8494309; doi:10.1371/journal.pone.0256989)
Supplement: Attachment — Submitted filename: Breslin Metabolic Cover letter for Revision v3..doc [file pone.0256989.s003.doc]

**Department of Nutritional Sciences •** New Brunswick Campus

| 65 Dudley Road  New Brunswick • New Jersey 08901  FAX: 848/932-6776 | Paul A.S. Breslin, Professor  848/932-6080 (office) 848/932-6081 (lab)  E-mail: breslin@SEBS.rutgers.edu |
| --- | --- |

05/17/2021

Dear Dr. John Glendinning:

I am submitting a revised manuscript [PONE-D-19-31346] titled: “Evidence that Human Oral Glucose Detection Involves A Sweet Taste Pathway and A Glucose Transporter Pathway.”

We have tried to address all of the editorial requests and the reviewer comments. In brief, between the two reviewers request for more data and experiments we have conducted four additional experiments and these data have been incorporated into the manuscript.

Experiment 1.

New Experiment to include fructose. Measure detection thresholds for 3 conditions: fructose alone, fructose with 20mM NaCl, fructose with phlorizin + 20mM Nacl. This addresses Reviewer #1 Major Comment #1 and Reviewer #2 Major Comments 1 & 2. This will determine whether SGLT proposed effects are specific to glucose.

Experiment 2.

New Experiment with MDG. Measure detection thresholds for 4 conditions: glucose alone, MDG alone, glucose with 20mM NaCl, MDG with 20mM Nacl - Reviewer #1 Major Comment 3. This will determine whether MDG can have enhanced signaling with NaCl via SGLT transport, but in the absence of increased metabolic signaling (ATP). We need to measure glucose along with MDG to see if the magnitude of NaCl enhancement is greater for glucose than for MDG, suggesting an additional metabolic signaling boost for glucose.

Experiment 3.

New Experiment investigating the effect of phlorizin on detection threshold of MDG. Measure detection threshold of MDG with 20 mM NaCl with and without phlorizin - Reviewer #1 Major Comment 3.

If SGLT enhances the signaling of MDG and NaCl, then phlorizin should prevent this.

Experiment 4.

New experiment with lactisole rinses to show that the potential "sweet water" taste produced by lactisole was taken into account - Reviewer #2 Major Comment 3.

Also, the editors requested we include additional information.

First, we provide here a revised **Competing Interests Statement**:

The authors declare that we have no competing interests in the work described here. The participation of the Suntory Global Innovation Center Limited does not alter our adherence to PLOS ONE policies on sharing data and materials.

And second, we provide a revised **Funding Statement**:

The funder did not play a role in the specific study design, data analyses, decision to publish, or preparation of the manuscript.

Third, we now include Excel Sheet that includes all of our original data for all figures presented.

Fourth, we now include an Ethics Statement in our methods section.

Fifth, all Figures are now referred to within the body of the text.

We address each individual reviewer's comments below.

Yours Sincerely,

Paul A.S. Breslin, Ph.D.

Professor, Nutritional Sciences, Rutgers &
Member, Monell Chemical Senses Center

Reviewer #1.

Major Comments:

1. Only glucose/sucralose detection thresholds were measured. Thus, it is not certain that SGLT1 signaling modulates sweet taste perception. In addition, "glucose comprises approximately half of the commercial sugar sweeteners used today" and glucose is much less sweet than fructose. Thus, it uncertain as to whether modulating SGLT1 activity with sodium chloride or phlorizin would alter the perceived sweetness of a HFCS-sweetened soft drink. The authors should consider including such an experiment.

We agree this is an interesting question. We are not studying suprathreshold perceived sweetness intensity in this paper. Rather, it has never been demonstrated that the human mouth can sense sugars via a nonT1R2-T1R3 mechanism. The first step here is to establish that this system is present and part of the sugar taste system. We will later determine the ultimate utility of this system for us and will include assays of sweet taste intensity, sugar preference, and sugar tolerance in oral challenges. But these are beyond the scope of the present manuscript.

2. The emphasis on oral "metabolic" sensing as opposed to glucose-sensing as contributing to the enhanced attraction to sugars is premature based on available rodent data. The finding that mice require the SUR1 component of the KATP sensing system but not T1R2-T1R3 taste receptors demonstrates that KATP metabolic sensing is required for the anticipatory insulin responses to glucose. However, SUR1 KO mice with impaired sugar KATP signaling are similar to WT mice in their avidity to glucose and sucrose in brief-access lick tests (Glendinning et al. 2017). Thus, oral metabolic sugar sensing does not appear to contribute to the attraction of mice to sugars.

We do not claim that this glucose detection mechanism is involved in attraction or reward, though this would be interesting to pursue in the future. And we have removed any conclusions about ATP-based metabolic sensing based on our new MDG experiment.

3. In addition to being similar to pancreatic beta cells, taste cells are also similar to intestinal enteroendocrine cells. This is relevant because SGLT1 acts as a glucose sensor in these cells in the release of various gut hormones. The nonmetabolizable SGLT-ligand alpha-methyl-D-glucose (MDG) has similar effects as glucose in promoting gut hormone release which demonstrates that glucose sensing not glucose metabolism is the critical event (Diakogiannaki et al. 2012, Physiol. Behav.). Mice (but not rats) prefer MDG to isomolar glucose in brief access choice tests (Ackroff & Sclafani, 2014, Physiol. Behav.). In addition, intragastric infusions of MDG, like glucose, conditions flavor preferences in mice (but not rats) (Ackroff & Sclafani, 2014; Zukerman et al., 2013, Am. J. Physiol. Regul. Integr. Comp. Physiol.). Together, these findings implicate SGLT1 sensing, not metabolic sensing in the attraction of mice to oral and postoral glucose and MDG. The authors should consider determining the effect of phlorizin on MDG detection and/or sweetness perception in humans. If phlorizin does NOT alter MDG detection/sweetness this would support the idea that glucose metabolism is critical. If phlorizin does alter MDG detection/sweetness this would indicate that SGLT1 glucose sensing is critical.

We have now tested MDG detection thresholds neat and with Sodium added and with phlorizin added. MDG thresholds are lowered by additional sodium and raised by the addition of phlorizin. This is an excellent suggestion and we feel that this experiment has clearly made the manuscript better, supporting the idea that SGLT transport is sufficient for signaling. We thank the reviewer for this suggestion. We also note that this does not preclude additional benefit of ATP signaling in the event that ATP can be generated.

4. The assertion that nonnutritive sweeteners are not as popular as sugars because of "oral sugar caloric signaling" is premature. Animal data indicate that postoral glucose signaling is the major reason why sugar (glucose and sucrose) are preferred to nonnutritive sweeteners. The evidence for postoral glucose reinforcement in humans is not as strong, but there are several reports that the postoral glucose (maltodextrin) enhances flavor preferences in humans.

Yes, you are clearly correct. We have now changed the language of the introduction to recognize a sugar signal post-orally that we now hypothesize also may originate from the within the mouth.

Additional Comments:

The Abstract statement that "Glucose has, therefore, been presumed to serve principally as a

sweetener in the mouth" is rather startling for two reasons. First, glucose is the least sweet of the common sugars, and glucose is obviously a critical nutrient for the brain. I recommend revising the statement to something like:

"Glucose, along with sucrose and fructose contributes to the sweet taste of foods."

We recognize the wording was awkward and have changed it per your recommendation.

Reviewer #2:

Major comments:

1. Comparing the effects of pharmacological treatments on the threshold for glucose solely to the threshold for a high potency sweetener (sucralose) is a significant weakness of the study design. The discussion in the Introduction of possible reasons why many consumers do not drink artificial sweeteners appears to have driven this choice. However, because the hypothesized SGLT pathway is selective to glucose, fructose offers the most logical, direct test of the overarching hypothesis. At a minimum, the omission of fructose needs to be explained.

We agree with this idea and have accordingly conducted a whole new study with fructose. Fructose is neither enhanced by the addition of sodium nor is it impeded by the addition of phlorizin, indicating the effects are very specific for glucose over fructose. This fructose study has improved the manuscript and we thank the reviewer for their comment. Curiously, the effects are seen with non-metabolizable glucose analog MDG which we also tested based on Reviewer 1 suggestion.

2. The importance of omitting fructose as a comparison stimulus is highlighted by the results of a prior study (Schiffman et al. 1999), which is not cited, that measured the inhibitory effects of Na-lactisole on the sweetness of 4 suprathreshold concentrations of glucose, fructose and 13 other sweeteners. The results showed that Na-lactisole completely blocked the sweetness of both monosaccharides at the 3 lower concentrations. In addition, a more recent paper by Pullicin et al. (2017), which also was not cited, reported that Na-lactisole prevented the discrimination of 75mM glucose from plain water. These studies must be cited and discussed as their results strongly imply that in humans, an SGLT pathway does not contribute significantly to the sweet taste of glucose. Say something about sweet taste and we are not argued that suprathreshold sweetness is determined by SGLT.

We agree that these are interesting and important papers. But the present work did not test the idea that the SGLT pathway contributes to sweetness intensity. This is not the point of this paper. In fact, as Reviewer 1 points out, glucose is only weakly sweet to begin with. Rather we set out to determine if the SGLT system in the mouth is functional at all in humans. The downstream implications of the SGLT system for suprathreshold sweetness, preference, liking, and glucose tolerance will all be studied at a later time.

3. The sweet water taste evoked by Na-lactisole (which occurs after expectoration and during water rinsing) was not mentioned as a possible confounding factor in experiment 1 and no safeguards were included in the procedure that might mitigate its possible effects on detection. This omission is surprising given the pioneering study on the sweet water taste that was conducted by some of the current authors (Galindo-Cuspinera et al, 2006). Their paper was cited only to support of using Na-lactisole to block T1R2-T1R3.

We agree this is an idea worth testing. We have, therefore, conducted a new experiment in which the sweet water taste was inhibited by including a small amount of Na Lactisole in the rinse water between stimuli. Under these circumstances the results we see are the same. These data are now included in a supplementary figure.

4. The authors state that a 100-fold difference in glucose and sucralose thresholds necessitated a log transformation of the data from all 3 experiments before statistical analysis. It is not immediately clear why a log transform was chosen. Were the thresholds for both glucose and sucralose distributed log-normally? In what ways did the log transform affect statistical outcomes in the 3 experiments? Why was transformation required to compare proportional (“fold”) differences in thresholds? Why were the untransformed data shown in the figures?

We no longer log transform the data and this is no longer an issue in the display of the data graphically either.

Additional comments:

1. Intro. Para. 1 last line: This statement implies that all sugars may engage a "second signaling pathway for calories" rather than specifically glucose.

We have modified the text of this sentence accordingly.

2. Intro. Para. 2, last sentence: The citation of Glendenning et al. 2015 omits the finding from that study that although T1R3 KO-mice showed a CPIR, they did not show a behavioral preference for glucose in water, a result consistent with the Schiffman et al 1999 data in humans. The Glendinning et al. study should be cited in the Discussion to consider the possibility that a SGLT mechanism may signal glucose in the mouth primarily through a metabolic pathway rather than through the sense of taste.

We have added this statement to the discussion.

3. Experiment 2, Stimulus and Materials: It should be noted in the Discussion that 20mM NaCl can be perceived as weakly sweet as well as weakly salty. Thus it cannot be assumed that the only mechanism for interaction with glucose and sucralose detection is augmentation of glucose transport is via a SGLT pathway.

It is true this is a known phenomenon. But this is not observed under all conditions. 20 mM NaCl can be perceived as sweet relative to adaptation to human saliva (One's own mouth), but when well adapted to plain water (rinsing out one's own saliva) it is rarely perceived as sweet. In the present work, subjects rinsed well with deionized water before each stimulus.

4. Because in Exps. 2 & 3 the psychophysical task required subjects to pick the solution that produced the “stronger” sensation, the procedure measured difference thresholds (JND’s) rather than detection thresholds.

This is a matter of semantics. We were not asking subjects to determine whether the two cups were the "same" or "different", which would be a threshold for difference, or a duo-trio test in which we ask the subject to identify which one is different.. Rather we wanted to know where the target is against a background. In some instances, the background was deionized Millipore filtered water (absolute detection threshold) and in other instances the background was the vehicle for phlorizin and NaCl. Regardless of the background, we always wanted to know where is the target indicated by perceived additional signal strength.

5. Discussion, next to last para: The speculation “…it may be possible to reduce sugar levels in beverages and foods by enhancing sugar metabolic signaling” overstates the potential importance of the present findings by failing to discuss the results of the psychophysical and behavioral studies that found no evidence in mice or humans of a significant contribution by a non-T1R2-T1R3 pathway to the sweetness of, or preference for, suprathreshold concentrations of glucose.

We cannot know for certain the potential of this system to influence preference or liking in humans, absent data. It is possible that this signal influences wanting or reward signaling for sugars. Furthermore, this comment presumes that glucose signaling has interpretive value as a solitary signal. In a food context it would always be accompanied by sweetness, textures, or aromas to which a signal, such as reward, could be associated. Although this comment is speculation and, therefore, may be incorrect, the findings of this manuscript nevertheless still hold this potential.
